# Mycobacteria induce TPL-2 mediated IL-10 in IL-4-generated alternatively activated macrophages

**DOI:** 10.1371/journal.pone.0179701

**Published:** 2017-06-28

**Authors:** Soumya Chatterjee, Kawsar R. Talaat, Emily E. van Seventer, Carl G. Feng, Alan L. Scott, Anne Jedlicka, Amanda Dziedzic, Thomas B. Nutman

**Affiliations:** 1 Laboratory of Parasitic Diseases, National Institute of Allergy and Infectious Diseases, National Institutes of Health, Bethesda, Maryland, United States of America; 2 Department of Molecular Microbiology and Immunology, Johns Hopkins Bloomberg School of Public Health, Baltimore, United States of America; Karolinska Institutet, SWEDEN

## Abstract

IL-4 drives expansion of Th2 cells that cause generation of alternatively activated macrophages (AAMs). Filarial infections are established early in life, induce increased IL-4 production are co-endemic with tuberculosis (TB). We sought to understand, therefore, how mycobacteria are handled in the context of IL-4-induced AAM. Comparing IL-4 generated in vitro monocyte derived human AAMs to LPS and IFN-γ generated classically macrophages (CAMs), both infected with mycobacteria (BCG), we demonstrated increased early BCG uptake and increased IL-10 production in AAMs compared to CAMs. We further demonstrated that increased IL-10 production is mediated by upregulation of tumor progression locus 2 (TPL-2), an upstream activator of extracellular signal related kinases (ERKs) in AAMs but not in CAMs, both at the transcript as well as the protein level. Pharmacologic inhibition of TPL-2 significantly diminished IL-10 production only in BCG-infected AAMs. Finally, we validated our findings in an *in vivo* C57Bl/6 model of filarial infection, where an exaggerated Th2 induced lung-specific alternative activation led to TPL-2 and IL-10 upregulation on subsequent TB infection. These data show that in response to mycobacterial infection, IL-4 generated AAMs in chronic filarial infections have impaired immune responses to TB infection by increasing IL-10 production in a TPL-2 mediated manner.

## Introduction

Filarial and other tissue invasive helminths have a widespread geographic distribution and affect more than 2 billion people in low-income, resource-limited areas of the world [1]. Exposure to these multicellular parasites often occurs early (in life) and repeatedly in most endemic areas [2]. These infections are characteristically chronic (as the lifespan of the adult parasites are often decades long). From an immunologic perspective in humans, early infection is characterized by a mixed Type1/Type2 response whose balance is altered at the time of patency (when egg laying or microfilariae appear) such that there is an expansion of IL-4 mediated Th2 responses [3, 4] which, over time, is modulated. Such modulation reflects an IL-10-dependent impairment of antigen-specific CD4+ T cell responsiveness. [3, 4] The expansion of Th2 cells (and the IL-4 produced) can, in turn, have profound effects on host monocytes and macrophages that result in systemic [5] as well as lung-specific alternative monocyte-macrophage activation [6–8].

Because helminth-endemic areas of the world are commonly co-endemic for *Mycobacterium tuberculosis* (Mtb) [9], the causative agent of tuberculosis, and because [10] macrophages are among the first lines of defense against Mtb [11] that may also determine the final outcome of the immune response to Mtb [12, 13], the state of activation of the macrophage becomes critical in the early containment of Mtb. Indeed, IFN-γ induced classical macrophage activation appears to be essential for mycobacterial killing through the induction of nitric oxide production and related reactive nitrogen intermediates (RNI) by macrophages through the action of inducible nitric oxide synthase (iNOS) [14]. In contrast, helminth infection-generated IL-4 induced, arginase (ARG-1) expressing alternative macrophage activation has been shown in murine systems to lead to impaired mycobacterial immunity [15].

In humans, however, the characterization of these macrophages has proved challenging, with inconsistent expression of iNOS and ARG-1 [16–18]. Additionally, the nature of the phagocytic activity and the responses of these macrophages against mycobacteria are not well defined.

Using cell surface markers of alternative macrophage activation [16, 17], we first sought to characterize the properties of human alternatively activated macrophages (AAM) and then compared these to classically activated macrophages (CAM) with particular emphasis on mycobacterial-induced responses. Our data provide strong evidence that human AAMs show enhanced susceptibility to mycobacterial infection, a susceptibility that was associated with the production of IL-10. In addition, we show that the mycobacterial induction of IL-10 is regulated by an IL-10 dependent feedback loop involving the MAP kinase kinase kinase tumor progression locus 2 (TPL-2). These findings were then confirmed in an in vivo murine model of filarial/Mtb co-infection in which we demonstrate that lung-specific alternative activation leads to a similar TPL-2-dependent IL-10 response.

## Materials and methods

### mf and BCG

*Live B*. *malayi* mf were obtained from the peritoneal cavities of infected gerbils (*Meriones unguiculatus*) or infected blood from cats under contract from the University of Georgia, Athens, GA. *Wild type BCG strain* Pasteur and BCG strain labeled with red fluorescent protein DsRed (BCG-RFP) were grown, quantified and stored as previously described [19].

### Cell cultures and infection

Healthy donor monocytes were cultured at 50 × 10^6^ per 6-well plate in serum-free media for 2 h, after which the medium was removed and complete media [RPMI 1640 medium (BioWhittaker) supplemented with 20 mM glutamine (BioWhittaker), 2% heat-inactivated human AB serum (Gemini Bioproducts), 100 IU/ml penicillin, and 100 g/ml streptomycin (Biofluids, Inc.)] was added. Monocytes were then cultured for 7 days with rhMCSF (50 ng/ml) (PeproTech Inc.), to generate macrophages. Subsequently, macrophage polarization was performed culturing with recombinant human IL-4 (rhIL-4; 50 ng/ml) for AAMs) or with LPS (1 μg/ml) (InvivoGen)/IFN-γ (20 ng/ml) for CAMs for 48 hours. For mf-activated macrophages, cells were exposed to live mf (50,000 per well) as previously described [20]. After 48h, the cells were harvested with Versene/EDTA (Biofluids Division, BioSource International), washed twice with phosphate-buffered saline (PBS; without Ca^2+^/Mg^2+^), counted by trypan blue exclusion. The average yield was 5X10^5^-1X10^6^ macrophages/condition. Cells were then re-cultured with live BCG at a multiplicity of infection (MOI) of 5. Cell and supernatant harvest was performed at 24 hours following exposure to infection with BCG for cytokine measurement. In another set of experiments, timed cell and supernatant harvest was performed pre-infection and 1, 4,6,18 and 24 hours post infection with BCG for both CAMs and AAMs.

### RNA preparation and real-time RT-PCR

Total RNA was prepared from 8 to 12 independent donors using an RNAEasy minikit (Qiagen). RNA (1 μg) from the cells was used to generate cDNA and then assessed by standard TaqMan assays (Applied Biosystems Inc.) as described previously [21]. The threshold cycle (CT), defined as the PCR cycle at which a statistically significant increase in reaction concentration is first detected, was calculated for the genes of interest and the 18S control and used to determine relative transcript levels.

Relative transcript levels were determined by the formula 1/ΔCT, where ΔCT is the difference between the CT of the target gene and that of the corresponding endogenous 18S reference.

### Western blot analysis for TPL-2 detection

Adherent cells were collected and RIPA lysis buffer (Santa Cruz Inc) was added to the cells. Cell lysates were boiled for 5 minutes; 25μL protein was run on a 1.5-mm 4% to 12% Tris gel and transferred onto PVDF membranes. After blocking using 5% nonfat milk for one hour, the membranes were incubated overnight at 4°C with either an antibody that detects an C-terminal epitope of TPL-2 (Cot [M-20], catalog # sc-720, Santa Cruz INC. with 1:500 dilution) or with rabbit anti- GAPDH (catalog # sc-25778, Santa Cruz INC. with 1:500 dilution). After washing, the membranes were incubated with HRP-conjugated anti—rabbit IgG (catalog # sc-2030, Santa Cruz INC. with 1:1000 dilution) at room temperature for 2 hours. The proteins were detected by chemiluminescence (luminol reagent, catalog # sc-2048, Santa Cruz INC). GAPDH was used as an internal control because of low background detection and a molecular weight distinct from the protein of interest in this study.

### Inhibition of IL-10 production using TPL-2 inhibitor

Increasing concentrations of the competitive TPL-2 inhibitor (4-(3-Chloro-4-fluorophenylamino)-6-(pyridin-3-yl-methylamino)-3-cyano- [1,7]-naphthyridine, C21H14ClFN6, EMD Millipore) were performed to determine the 50% inhibitory concentration (IC50) in AAMs and CAMs cell cultures for inhibition of IL-10 production. Following the experimental design mentioned above, BCG infected cells were treated with Tpl-2 inhibitor along with the same concentrations of vehicle (DMSO) used in parallel as controls. Supernatants were harvested at 24 hours post-infection for measurement of IL-10 by Luminex^™^.

### Quantifying western blot analysis

ImageJ (National Institutes of Health [NIH, Bethesda, MD] http://rsb.info.nih.gov/ij/) was used to quantify the intensity of the bands in the immunoblots.

### Cytokine and chemokine measurement

Cytokines (IL-6, IL-10, IL-12p40, IL-1α, IL-1β, and tumor necrosis factor alpha [TNF-α]) were assessed using a Milliplex Max human cytokine panel (Millipore, Billerica, MA). The minimum detectable concentration for the analytes was 3.2 pg/ml. For samples below the minimum detectable concentration of the assay, the value of 1 was assigned for analysis.

### Flow cytometry

Cell harvest and staining for flow cytometry was performed according to previously described protocols [22]. Analysis was performed using a FACSCanto II flow cytometer with FACSDiva software v.6 (Becton Dickinson). Antibodies used were fluorescein isothiocyanate (FITC)-conjugated mouse anti-human CD206 (BD Pharmingen) and APC-eFluor^®^ 780 conjugated mouse anti-human CD14.

Data were collected and analyzed using Flow Jo software (TreeStar Inc).

### Imagestream acquisition and analysis for assessment of mycobacterial uptake

Bacterial uptake was assessed using ImageStream^X^ Mk II (Amnis Corporation, Seattle, WA, USA). A 60x magnification was used for all samples. We harvested the AAMs and CAMs after 3 hours of infection with RFP labeled BCG at 5 MOI. A minimum of 10,000 cells were analyzed for each sample. Data analysis was performed using the IDEAS software (Amnis Corporation). A compensation matrix was generated using singly stained samples. The compensated data were then gated using the following approach. First, cells that were not in the field of focus were eliminated using Gradient RMS vs. Area features; next, macrophages were distinguished from free bacteria using the features of symmetry vs. circularity. Gating was then performed on RFP positive cells to identify macrophages associated with bacteria. The IDEAS software utilizes a feature called “Delta Centroid” that can accurately assess the distance between the center of two fluorescent probes and this feature was used to assess the distance between the center of the cell and the RFP labeled bacteria. An automated, standardized assessment of cells with internalized bacteria could therefore, be performed.

### Animals

C57BL/6 mice were purchased from Taconic Farms (Germantown, NY). Housing and breeding of all animals was done at the Association for the Assessment and Accreditation of Laboratory Animal Care-approved facility at the National Institute of Allergy and Infectious Diseases/National Institutes of Health according to the National Research Council Guide for the Care and Use of Laboratory Animals. All animals were maintained in AALAC-accredited BSL3 facilities at the NIH.

#### Mycobacterial and filarial infections of mice

Live B. *malayi* mf were obtained and purified as described above and intravenous injection of ~2.5X10^5^ mf was performed in the tail vein. For co-infection experiments, a total of 24 mice were utilized 19 mice were used for different infection experiments and 5 remained as uninfected controls.14 of 20 mice were initially made microfilaremic by i.v. injection in the tail vein. After 3 weeks, 9 of 14 mice were infected, using a previously well-established infection protocol [23]with ~100 CFU of the H37Rv strain of *M*. *tuberculosis* (Mtb) in a nose-only aerosol machine (CH Technologies) to generate filaria-Mtb co-infected mice (mf/Mtb) whereas the remaining 5 continued to be Mf positive (but Mtb-uninfected). Mtb infection in mf-unexposed mice was also performed in 5 mice. 4 weeks later, tissue harvest was performed in all four groups of mice (mf/mtb, mf and Mtb and uninfected controls).

#### Lung tissue harvest for qRT-PCR

Tissue harvest and RNA extraction for qRT-PCR was performed according to previously published protocols [8, 24, 25]

### Microarray methods

#### RNA quality

Quantitation was performed using a NanoDrop spectrophotometer and quality assessment was determined by RNA Nano LabChip analysis on an Agilent BioAnalyzer 2100.

One hundred nanograms of total RNA was processed for hybridization to Affymetrix Mouse Gene ST 1.0 microarrays using the Affymetrix GeneChip Whole Transcript Sense Target Labeling Assay Manual (http://media.affymetrix.com/support/downloads/manuals/wt_sensetarget_label_manual.pdf).

The signal amplification protocol for washing and staining of eukaryotic targets was performed in an automated fluidics station (Affymetrix FS450) using Affymetrix protocol FS450_0007. The arrays were scanned in the GCS3000 laser scanner with autoloader and 3G upgrade (Affymetrix). Quality assessment of hybridizations and scans was performed with Expression Console software (Affymetrix).

### Statistics

Data analyses were performed using GraphPad PRISM (GraphPad Software, Inc., San Diego, CA, USA). Median frequencies were used for measurements of central tendency. Statistically significant differences within groups were assessed by nonparametric Wilcoxon matched pairs signed rank test and between two groups were analyzed using the nonparametric Mann-Whitney U test. Kruskal-Wallis test with Dunn’s Multiple Comparison test was used for comparison among multiple groups.

#### Microarray statistical analysis

Partek Genomics Suite was used for detailed statistical analysis of microarray data. The RMA (Robust Multi-chip Average) Algorithm was used for background correction, normalization, and summarization of probes. Analysis of Variance (ANOVA) with linear contrasts was performed to generate p-values and fold changes; numerous plots (PCA, Volcano, Interaction, Dot, etc.); and creation and querying of gene lists.

### Study approval

CD14^+^ peripheral blood-derived monocytes were isolated from Leukopaks from healthy donors by counterflow centrifugal elutriation through Institutional Review Board (IRB)-approved protocols from the Department of Transfusion Medicine (Clinical Center, National Institutes of Health [NIH], Bethesda, MD). Mice were used according to an animal study proposal approved by the National Institute of Allergy and Infectious Diseases Animal Care and Use Committee.

## Results

### Characterization of human AAMs

**Fig 1:**
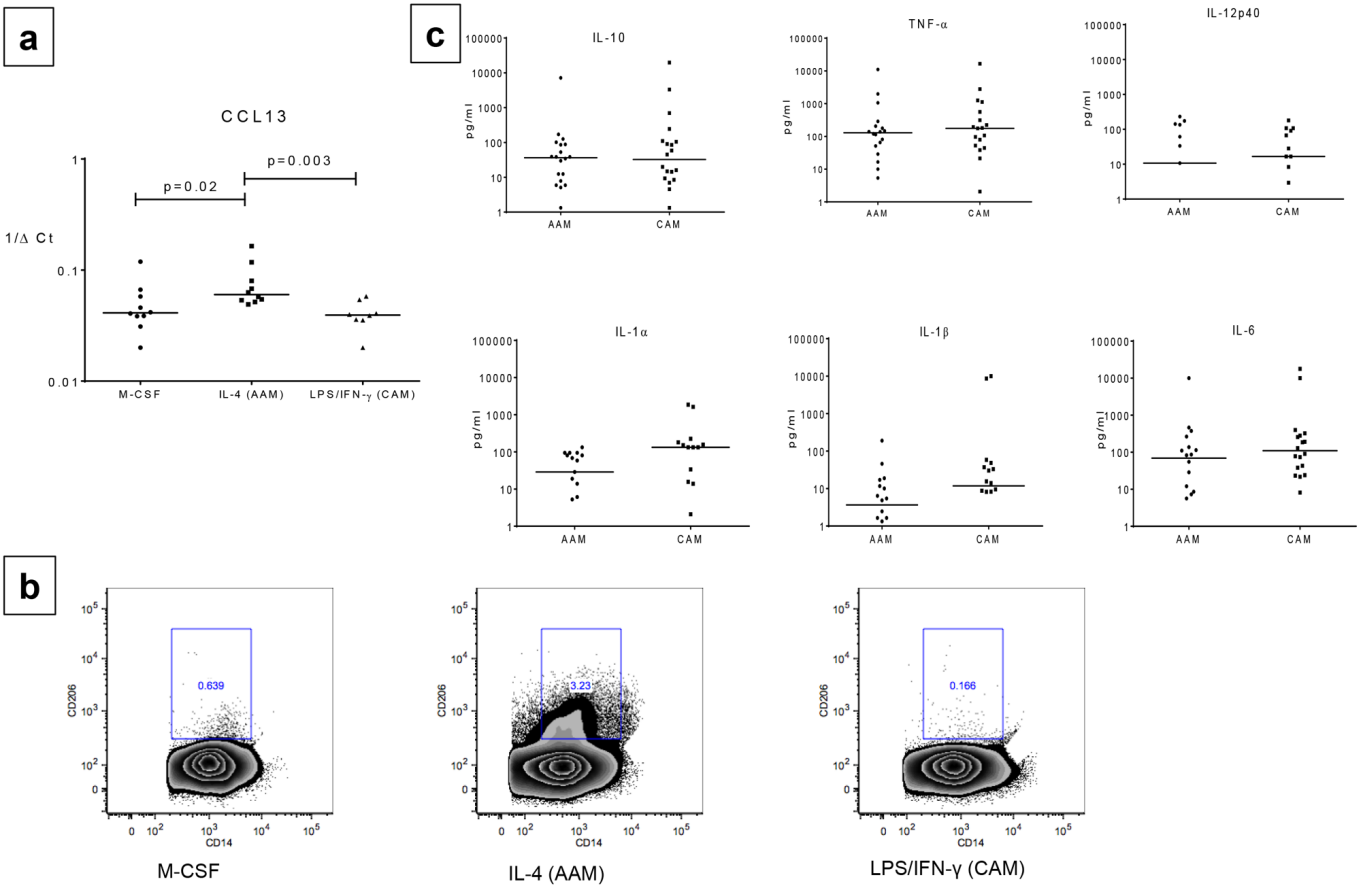
At baseline AAMs have increased CD206 (Mannose receptor) expression compared to CAMs. **Panel a**: Relative mRNA expression of previously defined AAM-specific marker (CCL13) expressed as 1/ΔCt, was compared between alternatively activated macrophages (AAMs) and classically activated macrophages (CAMs). Individual dots representing each subject. Horizontal bars represent median. **Panel b**: Representative plots showing CD206 (Mannose Receptor) expression (y-axis) compared on CD14+ cells (x-axis) between M-CSF,IL-4 (AAM) and LPS/IFN-γ (CAM) by flow cytometry **Panel c**:total cytokine production (IL-6, IL-10, IL-12p40, IL-1α, IL-1β, and tumor necrosis factor alpha [TNF-α]) in pg/ml is compared between AAM and CAM conditions. Cells were cultured with recombinant human IL-4 (rhIL-4; 50 ng/ml) for AAMs) or with LPS (1 μg/ml)/IFN-γ (20 ng/ml) for CAMs for 48 hours. Individual dots representing each subject. Horizontal bars represent median.

We first sought to delineate the unique characteristics of IL-4 generated AAMs compared to the LPS/IFN-γ generated CAMs. Although we were unable to detect consistently the expression of either iNOS or ARG1 by qRT-PCR in either of these 2 macrophage subsets (data not shown), we were able to demonstrate by consistent expression of CCL13 by AAMs (median 1/ΔCT = 0.06) compared to CAMs (median 1/ΔCT = 0.04, p = 0.02), (Fig 1a) though not of CCL17, CCL18, CCL22 or of IL-18,PDCDL1G, CD274, CLEC10A, CADH1 and CD274 (data not shown). Using flow cytometry, we next measured the expression of the mannose receptor (CD206), a well characterized marker of alternative activation, on the cell types generated by M-CSF (Mφs), IL-4 (AAMs) or LPS/IFN-γ (CAMs) by flow cytometry after gating initially on CD14+ cells (Fig 1b) and noted a significant increase in the frequency of cells expressing CD206 in the AAMs (median frequency [Fo] = 4.2%) compared to Mφs [Fo] = 0.72% or CAMs [Fo] = 0.13%, p = 0.03 (n = 8 donors). In addition, we assessed the pattern of cytokine production in supernatants and found no differences in IL-6, TNF-α, IL-12p40, IL-1 α, IL-1β or IL-10 levels between AAMs and CAMs (Fig 1c).

### AAMs, when compared to CAM show increased mycobacterial uptake

**Fig 2:**
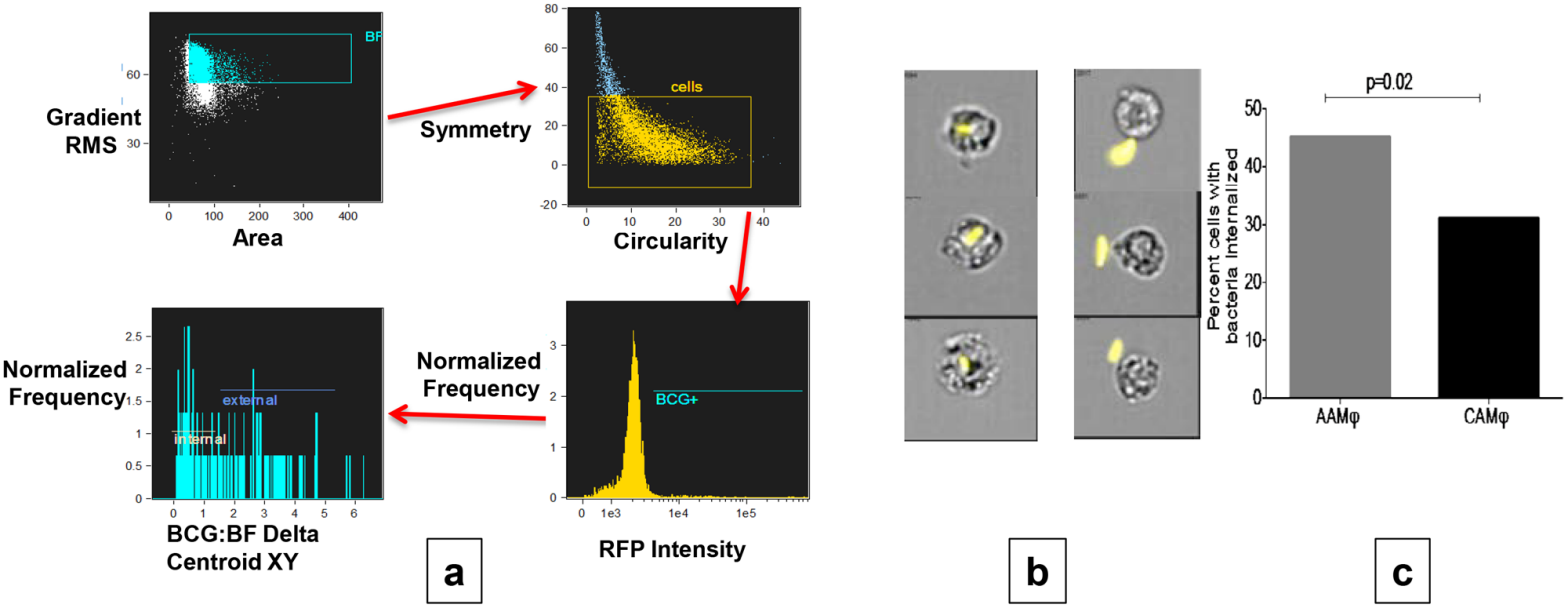
AAMs show increased BCG uptake compared to CAMs using ImageStream^X^. **Panel a** shows sequential gating strategy for assessing bacterial internalization within the cell. Cells that were in the field of focus were defined using Gradient RMS vs. Area features; next, plotting symmetry vs. circularity distinguished macrophages from free bacteria. Gating was then performed on RFP positive cells to identify macrophages associated with bacteria. Delta Centroid feature was used to assess the distance between the center of the cell and the RFP labeled bacteria to assess internalization. **Panel b**: representative images showing reproducibility and accuracy of internalization assessment using the above gating strategy. Three different examples of RFP labeled bacteria internalized (left panel) within macrophages vs. remaining external (right panel) are shown. **Panel c**: Graph comparing median percentage of cells internalizing bacteria (for n = 12 donors) using above strategy in AAMs (grey column) vs. CAMs (black column).

To assess uptake capabilities of the differentially polarized macrophages we harvested the AAMs and CAMs after 3 hours of infection with RFP labeled BCG at 5 MOI. Uptake was only assessed on cells with >90% viability using ImageStream^X^ Mk II from Amnis Corporation. Utilizing the gating strategy (described above in Methods, Fig 2a), the delta centroid feature provided a consistent estimate of percentage of cells with internalized bacteria (Fig 2b. As shown in Fig 2c, AAMs had a higher percentage of cells (median = 45%, minimum 43% and maximum 52%) with internalized bacteria compared to CAMs (median = 31%, minimum 30% and maximum 36%), p = 0.02.

### BCG infection decreases CCL13 expression and increases IL-10 production in AAMs but not in CAMs

**Fig 3:**
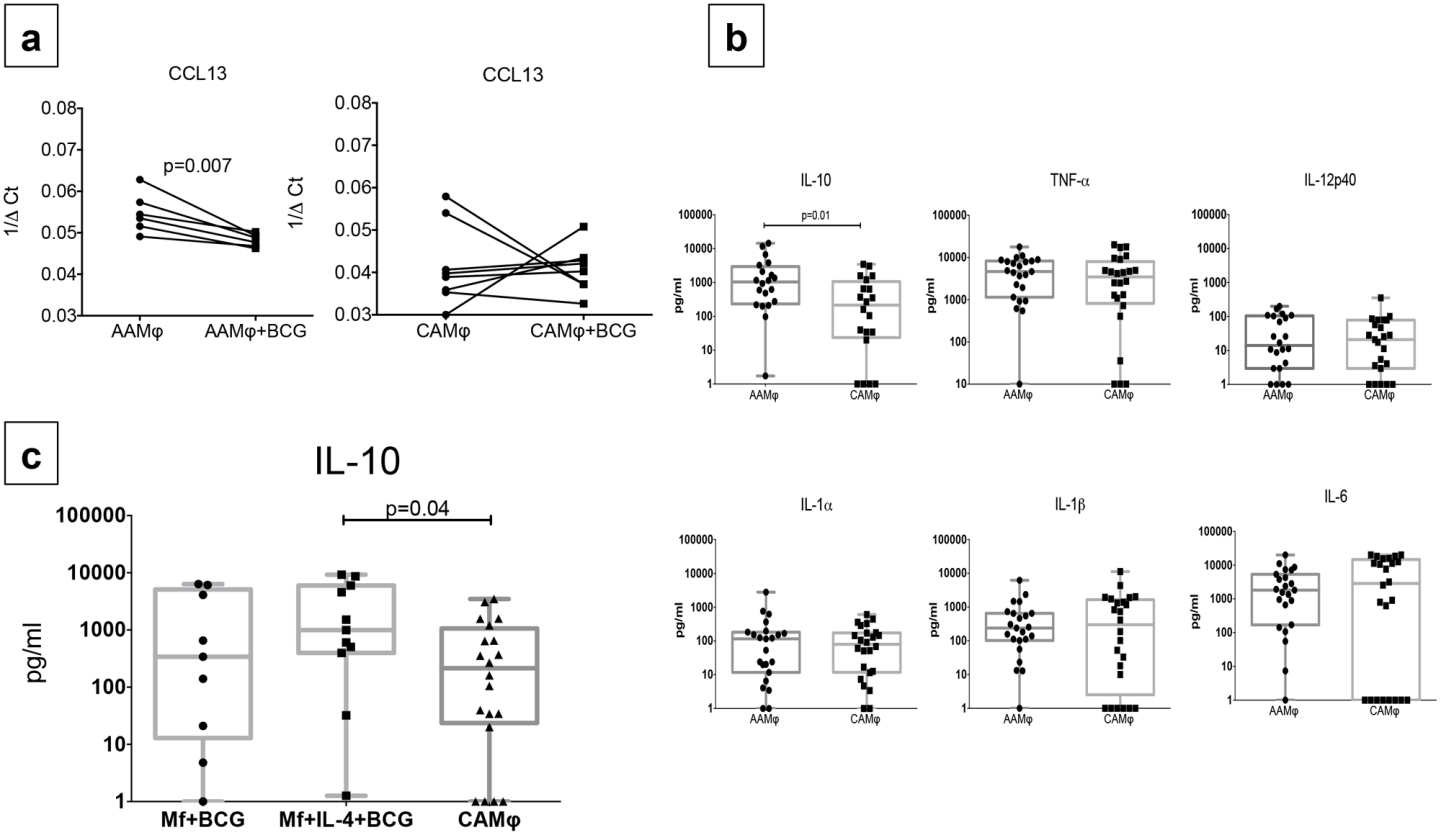
AAMs compared to CAMs show decreased CCL13 expression and increased IL-10 production post BCG infection. **Increased post-BCG IL-10 production was not seen in *B*. *malayi* microfilaria (mf) polarized macrophages. Panel a** shows change in relative mRNA expression of CCL13 from baseline to 24 hrs. post BCG infection expressed as 1/ΔCt in AAMs on the left panel and CAMs on the right panel. Individual lines represent each subject. **Panel b**: Net cytokine production (IL-6, IL-10, IL-12p40, IL-1α, IL-1β, and TNF-α) post BCG infection in pg/ml is compared between AAM and CAM conditions. Box and whisker plots represent median with 95% confidence intervals and individual dots representing each subject. **Panel c**: Net IL-10 production post BCG infection (measured in pg/ml) compared between mf polarized, mf+IL-4 polarized and CAMs. Box and whisker plots represent median with 95% confidence intervals and individual dots representing each subject.

We then sought to determine functional changes in response to BCG in AAMs and CAMs. Expression of the chemokine CCL13 after exposure to BCG was decreased compared to baseline in AAMs (median 1/ΔCT post BCG = 0.04, p = 0.007) but not in CAMs (median 1/ΔCT post BCG = 0.041) (Fig 3a). In addition, there was an approximately five fold increased net production of IL-10 was noted in AAMs (median net concentration = 1043 pg/ml) compared to CAMs (median net concentration = 214.5 pg/ml) (p = 0.01) on exposure to BCG. No difference was seen between the two macrophage subsets in the production of the other cytokines (Fig 3b).

To determine whether the increase in IL-10 after BCG infection was purely an effect of IL-4 generated AAMs or whether similar increase in IL-10 could also be elicited in macrophages pre-exposed to filarial parasites, M-CSF generated macrophages were cultured for 48 hours with mf of *B*.*malayi* alone or mf with IL-4 and contrasted the net production of IL-10 in these two conditions post-BCG with LPS/IFN-γ generated CAMs. While no differences were seen between mf polarized macrophages (median net concentration = 341 pg/ml) and CAMs (median net concentration = 214.5 pg/ml), we found a similar pattern of increased IL-10 production in the mf+IL-4 condition (median net concentration = 997.6 pg/ml) compared to CAMs, p = 0.04(Fig 3c), suggesting that the increased IL-10 production seen after BCG infection was a phenomenon specific to IL-4 rather than parasite generated induced alternative activation of macrophages.

### Increased IL-10 production in response to BCG occurs in AAMs by TPL-2 dependent mechanism

**Fig 4:**
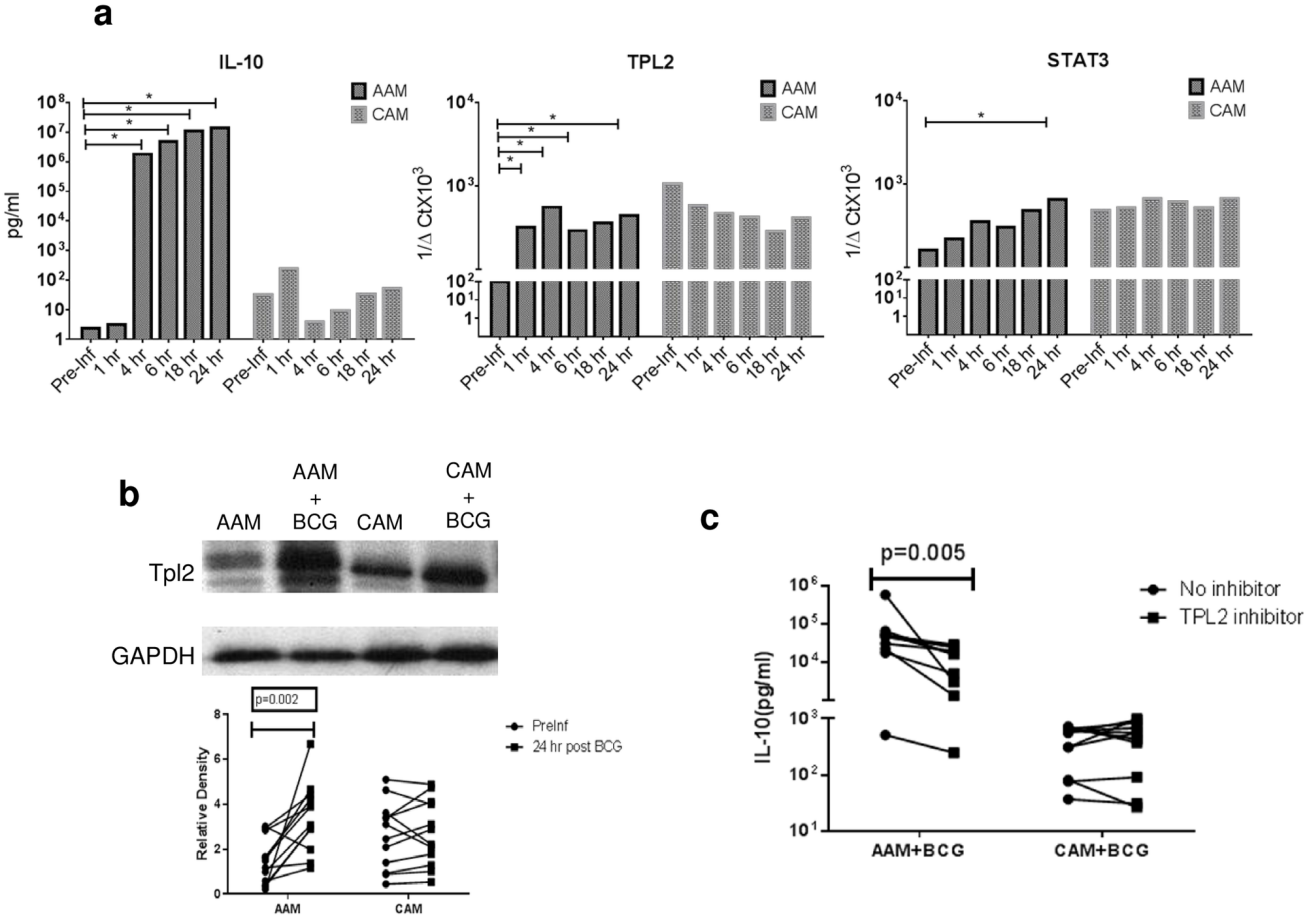
Increased IL-10 production in AAMs but not CAMs in is mediated by TPL-2. **Panel a**: Left panel (top and bottom) shows kinetics of net IL-10 production in AAMs and CAMs (in pg/ml) starting from pre BCG infection time point (pre-Inf) to 1, 4,6,18 and 24 hrs. post BCG infection. Middle panel shows relative mRNA expression of TPL-2 at the same time points, expressed as 1/ΔCt in AAMs and CAMs. Right panel shows relative mRNA expression of STAT3, expressed as 1/ΔCt in AAMs and CAMs. Vertical bars represent median relative expression Panel **b**: Whole cell extracts of AAMs and CAMs, generated at pre BCG infection and at 24hrs post infection were analyzed by immunoblotting with anti-TPL-2 antibodies utilizing anti-GAPDH as control (top panel). Bottom pane shows change in relative TPL-2 band density in AAMs vs. CAMs as assessed by ImageJ software comparing pre BCG infection time point (**Preinf**) with 24 hrs. post BCG infection (**24hr**). **Panel c**: Net IL-10 production (in pg/ml) is shown in AAMs and CAMs cultured with BCG alone for 24 hrs. or BCG and TPL-2 inhibitor (C_21_H_14_ClFN_6_) at 500nM (IC^50^). Individual dots representing each subject. A total of 12 subjects were analyzed for experiments in 4a, b and c.

To elucidate the mechanism of BCG infection induced increase in IL-10 production in the AAMs but not in the CAMs, the pre-infection IL-10 production (using Luminex^™^) and the relative mRNA expression (using qRT-PCR) of the important adaptor molecules involved in IL-10 regulation was contrasted with responses at 1,4,6,18 and 24 hours post infection within CAMs and AAMs. As shown in Fig 4a we noted an early (at 4hrs) and sustained increase in IL-10 production all donors (n = 6) in AAMs but not CAMs. Furthermore, as shown in S1 Table), an approximately 5-fold increased Toll-like Receptor 2 expression was noted in AAMs at 24 hrs. following BCG infection. No differences were seen between pre and 24 hr.-post infection time points for expression of the important molecules involved in Toll-like receptor (TLR) activated pathways of IL-10 production such as myeloid differentiation primary-response protein 88 (MYD88), extracellular signal regulated kinase (ERK), nuclear factor-κB (NF-κB) or p38 mitogen-activated protein kinases.

The expression of proteins involved in signal transducer and activator of transcription 3 (STAT3) dependent, IL-10 induced positive and negative feedback loops, dual specificity protein phosphatase 1 (DUSP1) and tumor progression locus 2 (TPL-2), respectively was examined next. Although, there was significantly decreased DUSP1 expression noted in CAMs post infection (S1 Table), an early and sustained relative upregulation of TPL-2 (Fig 4a) was seen in AAMs (p = 0.03 at 1,4,6 and 24 hours). This was associated with a relative upregulation of STAT3 at 24 hrs. following BCG infection in AAMs (p = 0.03) but not CAMs. As shown in Fig 4b, a significant increase in the level of TPL-2 protein was also detected by immunoblot at 24 hours following BCG infection in AAMs (median relative density pre-infection of 1.68 vs.5.71 post-infection, p = 0.03) but not in CAMs (median relative density pre-infection of 2.5 vs.3 post-infection), Finally, to determine the functional relationship between TPL-2 overexpression and IL-10 production by these macrophages, a competitive pharmacologic inhibitor of TPL-2 (C_21_H_14_ClFN_6_), was utilized, which displays significant selectivity for TPL-2 over other related kinases [26]. When BCG infected cells were cultured with 500nM of TPL-2 inhibitor (Fig 4c), the median percent inhibition of IL-10 production was 61.8% in AAMs and 27.8% in CAMs (p = 0.03).

### In vivo studies provide confirmation of alternative activation in filarial infection and show increased expression of TPL-2 associated with IL-10 upregulation

Given the lack of a permissive murine model of chronic filarial disease, we sought to confirm our in vitro findings at the tissue level in the C57BL/6 mouse model by establishing sustained microfilaremia for 7 weeks. We first measured mRNA expression of T-helper 1 (Th1) cytokines-IFN-γ and TNF-α and T-helper 2 (Th2) cytokines-IL-5 in the harvested lung tissues of the four different groups of mice—**uninfected**, microfilaremic only (**mf**), Mtb-infected only (**Mtb**) and co-infected mice that received Mtb infection three weeks after establishing microfilaremia (**mf/Mtb**). As expected, the **Mtb** group showed increased lung-specific relative expression of Th1 cytokines IFN-γ and TNF-α compared to both **uninfected** and **mf** (data not shown) groups. This was associated with increased iNOS-the marker defining murine macrophage classical activation. The reverse was seen in the **mf** group that showed an increase in IL-5 expression compared to **uninfected** and **Mtb** groups. Expression of macrophage markers of alternative activation (Chil3 chitinase-like 3, YM-1 and Mannose receptor, CD206) were increased in the mf group.

Interestingly, the mRNA expression profile in the **mf/Mtb** group mirrored that of the **Mtb** group both in terms of Th1 cytokine as well as markers of classical activation. Analysis of lung microarray data using the ANOVA data sheet created in Partek Genomics Suite demonstrated the highest expression of TPL-2 in the mf/Mtb group (fold change over uninfected control = 1.46, p = 0.00005). This was associated with increased IL-10 expression and was similar to our in vitro findings where we saw increased TPL-2 expression in mycobacteria infected AAMs. As before, when the fold increase in expression (compared to **uninfected** group), of the important molecules involved in TLR activated pathways of IL-10 regulation were contrasted between **mf/Mtb** and the **Mtb** group, similar increases were noted in both groups for TLR2, MyD88, Nucleotide-binding oligomerization domain-containing protein 2 (NOD2) and TNF receptor-associated factor 3 (TRAF3) (S2 Table). Decreased expression of Dual specificity protein phosphatase 1 (DUSP1), which is involved in the negative feedback loop for IL-10 production was noted in both groups. Despite increased TPL-2 expression there was no difference in mycobacterial load or lung pathology between the **Mtb** and **mf/Mtb** groups.

## Discussion

Macrophages are the first line cell defense against intracellular pathogens such as mycobacteria. It is now recognized that these cells exhibit considerable plasticity and their responses can be modulated by the ambient cytokine milieu. Chronic helminth infections (exposure to which occurs early in life in most endemic countries) lead to expansion of IL-4 mediated AAMs. Using a previously well characterized in vitro system of monocyte to macrophage differentiation [17] our study examines the differences in responses to mycobacteria by human AAMs (compared to CAMs) and show that AAMs are more susceptible to mycobacteria and produce increased amounts of mycobacterial-induced IL-10, a response enable by the activation of a positive feedback loop mediated by TPL-2. These findings were also paralleled in studies of Mtb/filarial co-infection in an in vivo mouse model.

Alternative macrophage activation has been defined differently in different studies, prompting a need for using standardized definitions [27]. We defined IL-4 polarized macrophages as alternatively activated in keeping with the original definition [28] and especially as chronic established filarial infections lead to expansion of IL-4 producing CD4+T cells [29]. This in turn, leads to alternative monocyte-macrophage activation [5]. Classical macrophages activated by a cytokine such as IFN-γ have been shown, historically, to produce pro-inflammatory cytokines such as IL-12 and IL-23 [30]. However, we saw no differences in baseline cytokine production between the two macrophage subsets in our in vitro system that might possibly reflect differences in activation conditions between the use of LPS/IFN-γ (used in the current study) and those driven by IFN-γ alone [31]. In addition, we found no differences in mRNA expression of ARG-1 and NOS-2 between human AAMs and CAMs (data not shown) consistent with what has been reported previously [16, 17].

Human AAM-specific chemokine and activation markers that have been previously characterized [17], but our data showed that CCL13 and CD206 were most often the major defining specific biomarkers of alternative activation. Exclusive CD206 expression AAMs in agreement with prior studies that have shown this receptor as consistently overexpressed in both human and mouse systems of IL-4 induced alternative activation [27, 32]. Interestingly, on exposure to BCG there was significantly diminished mRNA expression of CCL13, a potent chemokine known to attract CCR3+ cells such as eosinophils, Th2-lymphocytes, basophils. This finding is consistent with the our understanding of macrophage plasticity in which one or more functional properties of a particular macrophage phenotype can be altered following responses to different stimuli [33].

In this study we also established a standardized automated method for assessing mycobacterial uptake using ImageStream^X^ technology. This technique enables assessment of fluorescent protein labeled bacterial uptake in specific cell populations defined by flow cytometry using the “Delta Centroid” feature that provided accurate assessment of the uptake of RFP labeled bacteria. We saw increased mycobacterial uptake in AAMs but not CAMs. One of the possible explanations for this phenomenon could be up-regulation of the mannose receptor [34, 35] by AAMs, a receptor utilized by Mtb to gain entry into macrophages [36]. Engagement of the mannose receptor by Mtb has also been implicated in mycobacterial host evasion through upregulation of PPARγ expression [37] and by limiting phagosome-lysosome fusion [38]. There is no data available for differences in expression between AAMs and CAMs of other receptors like Complement receptor, surfactant protein A (SP-A) and SP-B, cholesterol in plasma membrane. Dendritic Cell-Specific ICAM-3-Grabbing Nonintegrin (DC-SIGN) expression is increased in macrophage cell lines in response to IL-4 [16]. This could be another mechanism by which enhanced uptake of mycobacteria occurs in AAMs. We did not test DC-SIGN expression in these experiments.

Increased bacterial survival has also been demonstrated in these AAMs due to IL-4- or IL-13-induced abrogation of autophagy [39] and has been suggested as a means by which there is increased mycobacterial survival. Our study also shows that IL-4 derived AAMs produce large amounts of IL-10 in response to mycobacterial infection, a response that is IL-4-specific given that this IL-10 upregulation was not seen following exposure to mf of B. malayi, a stimulus which has been reported to favor alternative activation [20].

The exact role of IL-10 in the context of Mtb infection is still being defined. It is however, well established that IL-10 blocks phagosome maturation [40] and production of reactive oxygen and nitrogen intermediates, both of which facilitate *Mtb* survival and outgrowth [41, 42]. High levels of IL-10 have been found both in the blood and serum of human subjects with pulmonary TB [43, 44] and reductions in bacterial load have now been shown in *Il10*^−/−^ mice on both the C57Bl/6 and BALB/c backgrounds as well as CBA/J mice that received sustained treatment of Mtb infection in the presence of an anti-IL-10R monoclonal antibody [45]. Thus, it can be hypothesized that early exaggerated IL-10 production as seen in the AAMs would be detrimental to pathogen clearance.

We also demonstrate that upregulation of the IL-10 activated feedback loop through STAT3 and TPL-2 is the mechanism by which AAMs produce IL-10 in response to mycobacterial infection. Interestingly, we were unable to find any differences in the TLR dependent MyD88 activated pathway of IL-10 induction between the two types of macrophages. The serine/threonine kinase TPL-2, also known as COT and MAP3K8, was independently discovered in the 1990s and identified initially as an oncogene [46]. TPL-2 is an upstream activator of ERK, and decreased ERK phosphorylation and IL-10 down-regulation has been shown previously in TPL-2^-/-^ macrophages after TLR activation [47]. It has also been shown, that IL-10 induces a highly restricted number of genes (including TPL-2) involved in the MAP kinase pathway in resting vs. activated macrophages [48] and a positive amplification loop in a STAT3 dependent manner, possibly through upregulation of TPL-2 as was seen in our study [49]. Finally, it was recently demonstrated that TPL-2/ERK-1/2 pathway was a positive regulator of TNF-a, IL-10, and IL-1b following Mtb infection of Macrophages and that regulation of IL-10 production through the TPL-2-ERK pathway and subsequent resistance to mycobacteria might depend on the presence of Type I interferons [50].

In this study, we were able to successfully establish microfilaremia in a C57Bl/6 model and demonstrate lung-specific upregulation of Th2 cytokine (IL-5) as well as previously well described murine markers of alternative activation [51]. Subsequent infection with Mtb resulted in a phenotype similar to Mtb infection alone with upregulation of Th1 cytokines and iNOS and downregulation of Th2 cytokines and YM-1 and CD206 consistent with what has been shown previously [15]. Although, the Th1 phenotype was dominant in the co-infected animals and we also did not see any difference in overall bacterial loads (between Mtb and mf/Mtb mice), our design might not be reflective of what might happen in the endemic setting where there is a high likelihood of parasitic reinfection leading to altered lung pathology [15]. Nevertheless, we noted significant mf-induced alternative activation in the lungs of mf-positive mice and significant IL-10 and TPL-2 upregulation in the lungs of the co-infected mice confirming our in vitro findings that background alternative macrophage activation leads to TPL-2 dependent IL-10 upregulation in the lung on subsequent Mtb infection. Chronic persistent helminth infection or reinfection may therefore lead to impaired mycobacterial immunity through sustained AAM generation and increased IL-10 production.

Our study helps further the definition of parasite infection generated, Th2 cytokine induced human alternative macrophage activation and defines a mechanism by which increased IL-10 production occurs in these cells after mycobacterial infection. These results have implications for understanding TB protective immunity and TB vaccine design in endemic areas where helminth infections are commonplace. Longitudinal studies of mycobacterial infection in blood and tissue derived monocyte-macrophage populations in helminth-infected subjects along with studies in animal models of chronic helminth infection are needed to further understand the immunomodulatory effects of helminth infections on TB.

## Supporting information

S1 Table Median relative gene expression (measured by q-RT-PCR and expressed as l/ΔCt xJ03l, of the important genes regulating IL-10 production in alternatively (AAM) and classically (CAM) activated macrophages. Pre-infection (Pre-Inf) with 24 hours post BCG infection time points are compared within each cell type. Increased TLR2 expression was seen in AAMs (and decreased DUSP l expression was noted in CAMs (Wilcoxon matched pairs signed rank test) Abbreviations: TLR, Toll-like Receptor; MYD88, Myeloid differentiation primary response gene 88;NFKB1, nuclear factor kappa-light-chain-enhancer of activated B cells; MAPK14 (p38), P38 mitogen-activated protein kinases; MAPK3 (ERK-I), extracellular-signal-regulated kinase l; MAPK l (ERK-2) extracellular-signal-regulated kinase 2;DUSPI, Dual specificity protein phosphatase l;GSK3B, Glycogen synthase kinase 3 beta; NOD2, Nucleotide-binding oligomerization domain-containing protein 2; TICAM I (TRIF),TIR-domain-containing adapter-inducing interferon-β;TRAF3, TNF receptor-associated factor 3.
(DOCX)

S2 Table Results of lung microarray comparing expression of the important genes regulating IL-10 production in filaria and *Mycobacterium tuberculosis* (Mtb) co-infected (mf/Mtb) mice and mice receiving Mtb infection only (Mtb). Data is expressed as fold increase vs. control (uninfected) mice (using the ANOVA data sheet created in Partek Genomics Suite). Increased TLR2, MyD88, NOD2 and TRAF3 expression with decreased expression of DUSP1 was seen in both groups while decreased ERK1 expression was noted in the mf/Mtb group only. Mtb group showed increased expression of NFKB1. Abbreviations: TLR, Toll-like Receptor; MYD88, Myeloid differentiation primary response gene 88;NFKB1, nuclear factor kappa-light-chain-enhancer of activated B cells; MAPK14 (p38), P38 mitogen-activated protein kinases; MAPK3 (ERK-1), extracellular-signal-regulated kinase 1; MAPK1 (ERK-2) extracellular-signal-regulated kinase 2;DUSP1, Dual specificity protein phosphatase 1;GSK3B, Glycogen synthase kinase 3 beta; NOD2, Nucleotide-binding oligomerization domain-containing protein 2; TICAM1 (TRIF),TIR-domain-containing adapter-inducing interferon-β;TRAF3, TNF receptor-associated factor 3.
(DOCX)

## References

[1] Hotez PJ, Brindley PJ, Bethony JM, King CH, Pearce EJ, Jacobson J. Helminth infections: the great neglected tropical diseases. J Clin Invest. 2008;118(4):1311–21. Epub 2008/04/03. doi: 10.1172/JCI34261 ; 18382743 10.1172/JCI34261 PMC2276811

[2] Hamlin KL, Moss DM, Priest JW, Roberts J, Kubofcik J, Gass K, et al Longitudinal Monitoring of the Development of Antifilarial Antibodies and Acquisition of Wuchereria bancrofti in a Highly Endemic Area of Haiti. PLoS Negl Trop Dis. 2012;6(12):e1941 doi: 10.1371/journal.pntd.0001941 23236534 10.1371/journal.pntd.0001941 PMC3516578

[3] Anuradha R, George PJ, Hanna LE, Chandrasekaran V, Kumaran P, Nutman TB, et al IL-4-, TGF-beta-, and IL-1-dependent expansion of parasite antigen-specific Th9 cells is associated with clinical pathology in human lymphatic filariasis. J Immunol. 2013;191(5):2466–73. Epub 2013/08/06. doi: 10.4049/jimmunol.1300911 ; 23913964 10.4049/jimmunol.1300911 PMC3764459

[4] Stewart GR, Boussinesq M, Coulson T, Elson L, Nutman T, Bradley JE. Onchocerciasis modulates the immune response to mycobacterial antigens. Clin Exp Immunol. 1999;117(3):517–23. Epub 1999/09/01. doi: 10.1046/j.1365-2249.1999.01015.x ; 10469056 10.1046/j.1365-2249.1999.01015.x PMC1905356

[5] Babu S, Kumaraswami V, Nutman TB. Alternatively activated and immunoregulatory monocytes in human filarial infections. J Infect Dis. 2009;199(12):1827–37. Epub 2009/05/22. doi: 10.1086/599090 ; 19456233 10.1086/599090 PMC3440875

[6] Pesce J, Kaviratne M, Ramalingam TR, Thompson RW, Urban JF, Cheever AW, et al The IL-21 receptor augments Th2 effector function and alternative macrophage activation. Journal of Clinical Investigation. 2006;116(7):2044–55. doi: 10.1172/JCI27727 16778988 10.1172/JCI27727 PMC1479424

[7] Porthouse KH, Chirgwin SR, Coleman SU, Taylor HW, Klei TR. Inflammatory responses to migrating Brugia pahangi third-stage larvae. Infect Immun. 2006;74(4):2366–72. Epub 2006/03/23. doi: 10.1128/IAI.74.4.2366-2372.2006 ; 16552066 10.1128/IAI.74.4.2366-2372.2006 PMC1418928

[8] Reece JJ, Siracusa MC, Scott AL. Innate immune responses to lung-stage helminth infection induce alternatively activated alveolar macrophages. Infect Immun. 2006;74(9):4970–81. Epub 2006/08/24. doi: 10.1128/IAI.00687-06 ; 16926388 10.1128/IAI.00687-06 PMC1594865

[9] Salgame P, Yap GS, Gause WC. Effect of helminth-induced immunity on infections with microbial pathogens. Nat Immunol. 2013;14(11):1118–26. Epub 2013/10/23. doi: 10.1038/ni.2736 . 24145791 10.1038/ni.2736 PMC4955540

[10] WHO. Global tuberculosis report 20142014. http://www.who.int/tb/publications/global_report/en/.

[11] Schlesinger LS. Role of mononuclear phagocytes in M tuberculosis pathogenesis. J Investig Med. 1996;44(6):312–23. Epub 1996/08/01. . 8795295

[12] Bloom BR, Flynn J, McDonough K, Kress Y, Chan J. Experimental approaches to mechanisms of protection and pathogenesis in M. tuberculosis infection. Immunobiology. 1994;191(4–5):526–36. Epub 1994/10/01. doi: 10.1016/S0171-2985(11)80459-6 . 7713567 10.1016/S0171-2985(11)80459-6

[13] Yoneda T, Ellner JJ. CD4(+) T cell and natural killer cell-dependent killing of Mycobacterium tuberculosis by human monocytes. Am J Respir Crit Care Med. 1998;158(2):395–403. Epub 1998/08/12. doi: 10.1164/ajrccm.158.2.9707102 . 9700112 10.1164/ajrccm.158.2.9707102

[14] Bonecini-Almeida MG, Lapa e Silva JR, Kritski AL, Neves Junior I, Morgado MG, Nathan C, et al Immune response during HIV and tuberculosis co-infection. Memorias do Instituto Oswaldo Cruz. 1998;93(3):399–402. Epub 1998/08/12. . 9698876 10.1590/s0074-02761998000300023

[15] Potian JA, Rafi W, Bhatt K, McBride A, Gause WC, Salgame P. Preexisting helminth infection induces inhibition of innate pulmonary anti-tuberculosis defense by engaging the IL-4 receptor pathway. The Journal of experimental medicine. 2011;208(9):1863–74. Epub 2011/08/10. doi: 10.1084/jem.20091473 ; 21825018 10.1084/jem.20091473 PMC3171086

[16] Semnani RT, Mahapatra L, Moore V, Sanprasert V, Nutman TB. Functional and Phenotypic Characteristics of Alternative Activation Induced in Human Monocytes by IL-4 or the Parasitic Nematode Brugia malayi. Infect Immun. 2011 Epub 2011/07/27. doi: 10.1128/IAI.05191-11 . 21788379 10.1128/IAI.05191-11 PMC3187236

[17] Martinez FO, Gordon S, Locati M, Mantovani A. Transcriptional profiling of the human monocyte-to-macrophage differentiation and polarization: new molecules and patterns of gene expression. J Immunol. 2006;177(10):7303–11. Epub 2006/11/04. . 17082649 10.4049/jimmunol.177.10.7303

[18] Murray PJ, Wynn TA. Obstacles and opportunities for understanding macrophage polarization. J Leukocyte Biol. 2011;89(4):557–63. doi: 10.1189/jlb.0710409 21248152 10.1189/jlb.0710409 PMC3058818

[19] Rothfuchs AG, Egen JG, Feng CG, Antonelli LR, Bafica A, Winter N, et al In situ IL-12/23p40 production during mycobacterial infection is sustained by CD11bhigh dendritic cells localized in tissue sites distinct from those harboring bacilli. J Immunol. 2009;182(11):6915–25. Epub 2009/05/21. doi: 10.4049/jimmunol.0900074 ; 19454688 10.4049/jimmunol.0900074 PMC2786988

[20] Semnani RT, Mahapatra L, Moore V, Sanprasert V, Nutman TB. Functional and phenotypic characteristics of alternative activation induced in human monocytes by interleukin-4 or the parasitic nematode Brugia malayi. Infect Immun. 2011;79(10):3957–65. Epub 2011/07/27. doi: 10.1128/IAI.05191-11 ; 21788379 10.1128/IAI.05191-11 PMC3187236

[21] Semnani RT, Keiser PB, Coulibaly YI, Keita F, Diallo AA, Traore D, et al Filaria-induced monocyte dysfunction and its reversal following treatment. Infect Immun. 2006;74(8):4409–17. Epub 2006/07/25. doi: 10.1128/IAI.01106-05 ; 16861626 10.1128/IAI.01106-05 PMC1539612

[22] Boyd A, Bennuru S, Wang Y, Sanprasert V, Law M, Chaussabel D, et al Quiescent innate response to infective filariae by human Langerhans cells suggests a strategy of immune evasion. Infect Immun. 2013;81(5):1420–9. Epub 2013/02/23. doi: 10.1128/IAI.01301-12 ; 23429540 10.1128/IAI.01301-12 PMC3648007

[23] Feng CG, Jankovic D, Kullberg M, Cheever A, Scanga CA, Hieny S, et al Maintenance of pulmonary Th1 effector function in chronic tuberculosis requires persistent IL-12 production. J Immunol. 2005;174(7):4185–92. Epub 2005/03/22. . 15778379 10.4049/jimmunol.174.7.4185

[24] Antonelli LR, Gigliotti Rothfuchs A, Goncalves R, Roffe E, Cheever AW, Bafica A, et al Intranasal Poly-IC treatment exacerbates tuberculosis in mice through the pulmonary recruitment of a pathogen-permissive monocyte/macrophage population. J Clin Invest. 2010;120(5):1674–82. Epub 2010/04/15. doi: 10.1172/JCI40817 ; 20389020 10.1172/JCI40817 PMC2860920

[25] Katz MS, Thatch KA, Schwartz MZ. Gene alterations and intestinal mucosal changes following growth factor and omega-3 exposure in a rat model of inflammatory bowel disease. Journal of pediatric surgery. 2013;48(2):345–52. Epub 2013/02/19. doi: 10.1016/j.jpedsurg.2012.11.013 . 23414863 10.1016/j.jpedsurg.2012.11.013

[26] Gavrin LK, Green N, Hu Y, Janz K, Kaila N, Li HQ, et al Inhibition of Tpl2 kinase and TNF-alpha production with 1,7-naphthyridine-3-carbonitriles: synthesis and structure-activity relationships. Bioorganic & medicinal chemistry letters. 2005;15(23):5288–92. Epub 2005/09/17. doi: 10.1016/j.bmcl.2005.08.029 . 16165349 10.1016/j.bmcl.2005.08.029

[27] Murray PJ, Allen JE, Biswas SK, Fisher EA, Gilroy DW, Goerdt S, et al Macrophage Activation and Polarization: Nomenclature and Experimental Guidelines. Immunity. 2014;41(1):14–20. Epub 2014/07/19. doi: 10.1016/j.immuni.2014.06.008 . 25035950 10.1016/j.immuni.2014.06.008 PMC4123412

[28] Stein M, Keshav S, Harris N, Gordon S. Interleukin 4 potently enhances murine macrophage mannose receptor activity: a marker of alternative immunologic macrophage activation. J Exp Med. 1992;176(1):287–92. Epub 1992/07/01. ; 1613462 10.1084/jem.176.1.287 PMC2119288

[29] Nutman TB, Kumaraswami V. Regulation of the immune response in lymphatic filariasis: perspectives on acute and chronic infection with Wuchereria bancrofti in South India. Parasite immunology. 2001;23(7):389–99. Epub 2001/07/27. . 11472558 10.1046/j.1365-3024.2001.00399.x

[30] Verreck FA, de Boer T, Langenberg DM, Hoeve MA, Kramer M, Vaisberg E, et al Human IL-23-producing type 1 macrophages promote but IL-10-producing type 2 macrophages subvert immunity to (myco)bacteria. Proceedings of the National Academy of Sciences of the United States of America. 2004;101(13):4560–5. Epub 2004/04/09. doi: 10.1073/pnas.0400983101 ; 15070757 10.1073/pnas.0400983101 PMC384786

[31] Nau GJ, Richmond JF, Schlesinger A, Jennings EG, Lander ES, Young RA. Human macrophage activation programs induced by bacterial pathogens. Proc Natl Acad Sci U S A. 2002;99(3):1503–8. Epub 2002/01/24. doi: 10.1073/pnas.022649799 ; 11805289 10.1073/pnas.022649799 PMC122220

[32] Martinez FO, Helming L, Milde R, Varin A, Melgert BN, Draijer C, et al Genetic programs expressed in resting and IL-4 alternatively activated mouse and human macrophages: similarities and differences. Blood. 2013;121(9):e57–69. Epub 2013/01/08. doi: 10.1182/blood-2012-06-436212 . 23293084 10.1182/blood-2012-06-436212

[33] Sica A, Mantovani A. Macrophage plasticity and polarization: in vivo veritas. J Clin Invest. 2012;122(3):787–95. Epub 2012/03/02. doi: 10.1172/JCI59643 ; 22378047 10.1172/JCI59643 PMC3287223

[34] Gordon S. Alternative activation of macrophages. Nat Rev Immunol. 2003;3(1):23–35. Epub 2003/01/04. doi: 10.1038/nri978 . 12511873 10.1038/nri978

[35] Mosser DM. The many faces of macrophage activation. J Leukoc Biol. 2003;73(2):209–12. Epub 2003/01/30. . 12554797 10.1189/jlb.0602325

[36] Schlesinger LS. Macrophage phagocytosis of virulent but not attenuated strains of Mycobacterium tuberculosis is mediated by mannose receptors in addition to complement receptors. J Immunol. 1993;150(7):2920–30. Epub 1993/04/01. . 8454864

[37] Rajaram MV, Brooks MN, Morris JD, Torrelles JB, Azad AK, Schlesinger LS. Mycobacterium tuberculosis activates human macrophage peroxisome proliferator-activated receptor gamma linking mannose receptor recognition to regulation of immune responses. J Immunol. 2010;185(2):929–42. Epub 2010/06/18. doi: 10.4049/jimmunol.1000866 ; 20554962 10.4049/jimmunol.1000866 PMC3014549

[38] Kang PB, Azad AK, Torrelles JB, Kaufman TM, Beharka A, Tibesar E, et al The human macrophage mannose receptor directs Mycobacterium tuberculosis lipoarabinomannan-mediated phagosome biogenesis. J Exp Med. 2005;202(7):987–99. Epub 2005/10/06. doi: 10.1084/jem.20051239 ; 16203868 10.1084/jem.20051239 PMC2213176

[39] Harris J, De Haro SA, Master SS, Keane J, Roberts EA, Delgado M, et al T helper 2 cytokines inhibit autophagic control of intracellular Mycobacterium tuberculosis. Immunity. 2007;27(3):505–17. Epub 2007/09/26. doi: 10.1016/j.immuni.2007.07.022 . 17892853 10.1016/j.immuni.2007.07.022

[40] O'Leary S, O'Sullivan MP, Keane J. IL-10 blocks phagosome maturation in mycobacterium tuberculosis-infected human macrophages. Am J Respir Cell Mol Biol. 2011;45(1):172–80. Epub 2010/10/05. doi: 10.1165/rcmb.2010-0319OC . 20889800 10.1165/rcmb.2010-0319OC

[41] Gazzinelli RT, Oswald IP, James SL, Sher A. IL-10 inhibits parasite killing and nitrogen oxide production by IFN-gamma-activated macrophages. J Immunol. 1992;148(6):1792–6. Epub 1992/03/15. . 1541819

[42] Redpath S, Ghazal P, Gascoigne NR. Hijacking and exploitation of IL-10 by intracellular pathogens. Trends in microbiology. 2001;9(2):86–92. Epub 2001/02/15. . 11173248 10.1016/s0966-842x(00)01919-3

[43] Almeida AS, Lago PM, Boechat N, Huard RC, Lazzarini LC, Santos AR, et al Tuberculosis is associated with a down-modulatory lung immune response that impairs Th1-type immunity. J Immunol. 2009;183(1):718–31. Epub 2009/06/19. doi: 10.4049/jimmunol.0801212 . 19535630 10.4049/jimmunol.0801212

[44] Barnes PF, Lu S, Abrams JS, Wang E, Yamamura M, Modlin RL. Cytokine production at the site of disease in human tuberculosis. Infect Immun. 1993;61(8):3482–9. Epub 1993/08/01. ; 8335379 10.1128/iai.61.8.3482-3489.1993 PMC281026

[45] Redford PS, Boonstra A, Read S, Pitt J, Graham C, Stavropoulos E, et al Enhanced protection to Mycobacterium tuberculosis infection in IL-10-deficient mice is accompanied by early and enhanced Th1 responses in the lung. Eur J Immunol. 2010;40(8):2200–10. Epub 2010/06/03. doi: 10.1002/eji.201040433 ; 20518032 10.1002/eji.201040433 PMC3378704

[46] Miyoshi J, Higashi T, Mukai H, Ohuchi T, Kakunaga T. Structure and transforming potential of the human cot oncogene encoding a putative protein kinase. Molecular and cellular biology. 1991;11(8):4088–96. Epub 1991/08/01. ; 2072910 10.1128/mcb.11.8.4088-4096.1991 PMC361219

[47] Kaiser F, Cook D, Papoutsopoulou S, Rajsbaum R, Wu X, Yang HT, et al TPL-2 negatively regulates interferon-beta production in macrophages and myeloid dendritic cells. J Exp Med. 2009;206(9):1863–71. Epub 2009/08/12. doi: 10.1084/jem.20091059 ; 19667062 10.1084/jem.20091059 PMC2737152

[48] Lang R, Patel D, Morris JJ, Rutschman RL, Murray PJ. Shaping gene expression in activated and resting primary macrophages by IL-10. J Immunol. 2002;169(5):2253–63. Epub 2002/08/24. . 12193690 10.4049/jimmunol.169.5.2253

[49] Staples KJ, Smallie T, Williams LM, Foey A, Burke B, Foxwell BM, et al IL-10 induces IL-10 in primary human monocyte-derived macrophages via the transcription factor Stat3. J Immunol. 2007;178(8):4779–85. Epub 2007/04/04. . 17404258 10.4049/jimmunol.178.8.4779

[50] McNab FW, Ewbank J, Rajsbaum R, Stavropoulos E, Martirosyan A, Redford PS, et al TPL-2-ERK1/2 signaling promotes host resistance against intracellular bacterial infection by negative regulation of type I IFN production. J Immunol. 2013;191(4):1732–43. Epub 2013/07/12. doi: 10.4049/jimmunol.1300146 ; 23842752 10.4049/jimmunol.1300146 PMC3796877

[51] Mosser DM, Edwards JP. Exploring the full spectrum of macrophage activation. Nat Rev Immunol. 2008;8(12):958–69. Epub 2008/11/26. doi: 10.1038/nri2448 . 19029990 10.1038/nri2448 PMC2724991

