# Supplementary material for: Mycobacteria induce TPL-2 mediated IL-10 in IL-4-generated alternatively activated macrophages
Source: PLoS One. 2017 Jun 28;12(6):e0179701. doi: 10.1371/journal.pone.0179701 (PMC5489173; doi:10.1371/journal.pone.0179701)
Supplement: S1 Table — Pre-infection (Pre-Inf) with 24 hours post BCG infection time points are compared within each cell type. Increased TLR2 expression was seen in AAMs (and decreased DUSP l expression was noted in CAMs (Wilcoxon matched pairs signed rank test) Abbreviations: TLR, Toll-like Receptor; MYD88, Myeloid differentiation primary response gene 88;NFKB1, nuclear factor kappa-light-chain-enhancer of activated B cells; MAPK14 (p38), P38 mitogen-activated protein kinases; MAPK3 (ERK-I), extracellular-signal-regulated kinase l; MAPK l (ERK-2) extracellular-signal-regulated kinase 2;DUSPI, Dual specificity protein phosphatase l;GSK3B, Glycogen synthase kinase 3 beta; NOD2, Nucleotide-binding oligomerization domain-containing protein 2; TICAM I (TRIF),TIR-domain-containing adapter-inducing interferon-β;TRAF3, TNF receptor-associated factor 3. (DOCX) [file pone.0179701.s001.docx]

**S1 Table**

Median relative gene expression, (measured by q-RT-PCR and expressed as1/ΔCt x10^3)^, of the important genes regulating IL-10 production in alternatively (AAM) and classically (CAM) activated macrophages. Pre-infection (Pre-Inf) with 24 hours post BCG infection time points are compared within each cell type.

Increased TLR2 expression was seen in AAMs (and decreased DUSP1 expression was noted in CAMs (Wilcoxon matched pairs signed rank test)

Abbreviations: TLR, Toll-like Receptor; MYD88, Myeloid differentiation primary response gene 88;NFKB1, nuclear factor kappa-light-chain-enhancer of activated B cells; MAPK14 (p38), P38 mitogen-activated protein kinases; MAPK3 (ERK-1), extracellular-signal-regulated kinase 1; MAPK1 (ERK-2) extracellular-signal-regulated kinase 2;DUSP1, Dual specificity protein phosphatase 1;GSK3B, Glycogen synthase kinase 3 beta; NOD2, Nucleotide-binding oligomerization domain-containing protein 2; TICAM1 (TRIF),TIR-domain-containing adapter-inducing interferon-β;TRAF3, TNF receptor-associated factor 3
