# Supplementary material for: Mycobacteria induce TPL-2 mediated IL-10 in IL-4-generated alternatively activated macrophages
Source: PLoS One. 2017 Jun 28;12(6):e0179701. doi: 10.1371/journal.pone.0179701 (PMC5489173; doi:10.1371/journal.pone.0179701)
Supplement: S2 Table — Data is expressed as fold increase vs. control (uninfected) mice (using the ANOVA data sheet created in Partek Genomics Suite). Increased TLR2, MyD88, NOD2 and TRAF3 expression with decreased expression of DUSP1 was seen in both groups while decreased ERK1 expression was noted in the mf/Mtb group only. Mtb group showed increased expression of NFKB1. Abbreviations: TLR, Toll-like Receptor; MYD88, Myeloid differentiation primary response gene 88;NFKB1, nuclear factor kappa-light-chain-enhancer of activated B cells; MAPK14 (p38), P38 mitogen-activated protein kinases; MAPK3 (ERK-1), extracellular-signal-regulated kinase 1; MAPK1 (ERK-2) extracellular-signal-regulated kinase 2;DUSP1, Dual specificity protein phosphatase 1;GSK3B, Glycogen synthase kinase 3 beta; NOD2, Nucleotide-binding oligomerization domain-containing protein 2; TICAM1 (TRIF),TIR-domain-containing adapter-inducing interferon-β;TRAF3, TNF receptor-associated factor 3. (DOCX) [file pone.0179701.s002.docx]

**S2 Table**

Results of lung microarray comparing expression of the important genes regulating IL-10 production in filaria and *Mycobacterium tuberculosis* (Mtb) co-infected (**mf/Mtb**) mice and mice receiving Mtb infection only (**Mtb**). Data is expressed as fold increase vs. control (**uninfected**) mice (using the ANOVA data sheet created in Partek Genomics Suite). Increased TLR2, MyD88, NOD2 and TRAF3 expression with decreased expression of DUSP1 was seen in both groups while decreased ERK1 expression was noted in the **mf/Mtb** group only. **Mtb** group showed increased expression of NFKB1.

Abbreviations: TLR, Toll-like Receptor; MYD88, Myeloid differentiation primary response gene 88;NFKB1, nuclear factor kappa-light-chain-enhancer of activated B cells; MAPK14 (p38), P38 mitogen-activated protein kinases; MAPK3 (ERK-1), extracellular-signal-regulated kinase 1; MAPK1 (ERK-2) extracellular-signal-regulated kinase 2;DUSP1, Dual specificity protein phosphatase 1;GSK3B, Glycogen synthase kinase 3 beta; NOD2, Nucleotide-binding oligomerization domain-containing protein 2; TICAM1 (TRIF),TIR-domain-containing adapter-inducing interferon-β;TRAF3, TNF receptor-associated factor 3
